# Electroacupuncture exerts prolonged analgesic and neuroprotective effects in a persistent dental pain model induced by multiple dental pulp injuries: GABAergic interneurons-astrocytes interaction

**DOI:** 10.3389/fimmu.2023.1213710

**Published:** 2023-10-26

**Authors:** Sharmely Sharon Ballon Romero, Lih-Jyh Fuh, Shih-Ya Hung, Yu-Chen Lee, Yu-Chuen Huang, Szu-Yu Chien, Yi-Hung Chen

**Affiliations:** ^1^ Graduate Institute of Acupuncture Science, China Medical University, Taichung, Taiwan; ^2^ School of Dentistry, College of Dentistry, China Medical University, Taichung, Taiwan; ^3^ Division of Surgery, Department of Medical Research, China Medical University Hospital, Taichung, Taiwan; ^4^ Department of Acupuncture, China Medical University Hospital, Taichung, Taiwan; ^5^ Department of Medical Research, China Medical University Hospital, School of Chinese Medicine, China Medical University, Taichung, Taiwan; ^6^ Chinese Medicine Research Center, China Medical University, Taichung, Taiwan; ^7^ Department of Photonics and Communication Engineering, Asia University, Taichung, Taiwan

**Keywords:** dental pulp injury, astrocytes, glutamate transporter-1, glutamine synthetase, GABAergic interneurons, electroacupuncture

## Abstract

Pain within the trigeminal system, particularly dental pain, is poorly understood. This study aimed to determine whether single or multiple dental pulp injuries induce persistent pain, its association with trigeminal central nociceptive pathways and whether electroacupuncture (EA) provides prolonged analgesic and neuroprotective effects in a persistent dental pain model. Models of single dental pulp injury (SDPI) and multiple dental pulp injuries (MDPI) were used to induce trigeminal neuropathic pain. The signs of dental pain-related behavior were assessed using the mechanical head withdrawal threshold (HWT). Immunofluorescence and western blot protocols were used to monitor astrocyte activation, changes in apoptosis-related proteins, and GABAergic interneuron plasticity. SDPI mice exhibited an initial marked decrease in HWT from days one to 14, followed by progressive recovery from days 21 to 42. From days 49 to 70, the HWT increased and returned to the control values. In contrast, MDPI mice showed a persistent decrease in HWT from days one to 70. MDPI increased glial fibrillary acidic protein (GFAP) and decreased glutamine synthetase (GS) and glutamate transporter-1 (GLT1) expression in the Vi/Vc transition zone of the brainstem on day 70, whereas no changes in astrocytic markers were observed on day 70 after SDPI. Increased expression of cleaved cysteine-aspartic protease-3 (cleaved caspase-3) and Bcl-2-associated X protein (Bax), along with decreased B-cell lymphoma/leukemia 2 (Bcl-2), were observed at day 70 after MDPI but not after SDPI. The downregulation of glutamic acid decarboxylase (GAD65) expression was observed on day 70 only after MDPI. The effects of MDPI-induced lower HWT from days one to 70 were attenuated by 12 sessions of EA treatment (days one to 21 after MDPI). Changes in astrocytic GFAP, GS, and GLT-1, along with cleaved caspase-3, Bax, Bcl-2, and GAD65 expression observed 70 days after MDPI, were reversed by EA treatment. The results suggest that persistent dental pain in mice was induced by MDPI but not by SDPI. This effect was associated with trigeminal GABAergic interneuron plasticity along with morphological and functional changes in astrocytes. EA exerts prolonged analgesic and neuroprotective effects that might be associated with the modulation of neuron-glia crosstalk mechanisms.

## 1 Introduction

Pain originating from the teeth or their supporting structures, also known as “dental pain” or toothache, is one of the most common types of orofacial conditions (1). This unique kind of pain experienced by adults and children is described as “the highest level possible” (2). Dental pain is associated with inflammation and infection of the dental pulp and usually occurs due to untreated caries or dental trauma (3). The clinical diagnosis defines mild to moderate dental pulp inflammation as reversible pulpitis, which usually resolves once the etiology is removed (4). However, in irreversible pulpitis, damage to the dental pulp goes beyond self-repair and eventually becomes necrotic (4). Despite the treatment aimed at relieving pain, such as filling, root canal, or even extraction, dental pain can persist and lead to lasting consequences to the trigeminal afferents that innervate the dental pulp (5–7). However, the chronicity of dental pain remains poorly documented (8). The current data available estimates that patients have persistent toothache for about 27.6 or 55.2 days before they seek emergency dental treatment (9, 10). Despite its high worldwide prevalence (9), no experimental information associated with persistent dental pain is available.

Previous studies used the dental pulp injury model type III (DPI) introduced by Byers to reproduce the clinical irreversible pulpitis profile in rodents (11). This model involves mechanical exposure of the dental pulp of a single molar (12). However, following Byers method, only transient behavioral and molecular outcomes have been reported by ourselves and other groups (5, 6, 13–16). Hence, in this study, we aimed to develop a more effective animal model of persistent dental pain induced by multiple-spaced dental pulp injuries (MDPI).

Astrocytes are the most abundant cell type in the brain and play an essential role in a wide variety of crucial functions in healthy or pathological conditions of the central nervous system (CNS) (17). Astrocytes form an interconnected network via intercellular channels (gap junctions), which allows them to support neurons and control the surrounding environment during synaptic transmission through astrocytic glutamate transporters (18). The astrocytic uptake of extracellular glutamate and γ-aminobutyric acid (GABA) from the synaptic cleft through glutamate transporter type-1 (GLT-1), the major glutamate transporter, is then metabolized by glutamine synthetase (GS) to glutamine (19, 20). This synthesized glutamine, a specific function of astrocytes, is released to the synaptic space and subsequently taken up by glutamatergic and GABAergic neurons for future synthesis of either glutamate or GABA (21). Therefore, astrocytic GLT-1 and GS also prevent glutamate excitotoxicity by clearing glutamate from the synaptic cleft (22). Moreover, in response to exacerbated conditions, such as inflammation and pain, astrocytes undergo morphological and metabolic shifts (from resting to an activated state) mediated by the increased release of adenosine triphosphate (ATP), glutamate and chemokines released from neurons (23). Astrocyte reactive states are increasingly recognized as the culprit of alterations in glutamate metabolism that play a critical role in the development and maintenance of chronic pain (24). Excessive release of glutamate induces significant changes in mitochondrial functions, resulting in increased reactive oxygen species generation (25), which triggers changes in key apoptosis-related proteins, such as cysteine-aspartic acid protease-3 (Caspase-3), Bcl-2 associated protein X (Bax), and B-cell lymphoma/leukemia 2 (Bcl-2), which ultimately could lead to the development of hypersensitivity and neuronal death (26, 27). In addition, astrocyte reactivity may affect the homeostasis of the glutamate-glutamine cycle. Since astrocytes modulate GABAergic transmission through different mechanisms (28), they may also cause an imbalance between excitatory glutamatergic and inhibitory GABAergic transmission (29). In contrast, functional plasticity in the GABAergic system is also involved in central sensitization (30). However, the implications of these crucial CNS mechanisms involving persistent dental pain remain unclear.

Pain management is one of the most challenging goals of dentists. Conventional analgesic medications, such as nonsteroidal anti-inflammatory drugs (NSAIDs), are an integral part of dental practice (31). However, in patients where no local source of pathology can be found, but the symptomatology persists, there is an increased risk of developing unpleasant side effects, or the therapy itself might be ineffective over time (32). Therefore, a multidisciplinary approach is necessary. Accordingly, manual acupuncture (MA) or electroacupuncture (EA) is one of the best-known complementary and alternative therapies for pain management (33). Interestingly, mounting scientific evidence suggests that acupuncture is effective in controlling preoperative and postoperative orofacial pain (34, 35). Furthermore, the World Health Organization (WHO) has recognized the use of acupuncture for several symptoms, diseases, and conditions, including orofacial pain (36).

Therefore, this study aimed to identify whether persistent dental pain induced by MDPI is associated with astrocyte reactivity in the trigeminal subnuclei interpolaris/caudalis (Vi/Vc) transition zone of the brainstem as well as apoptotic signaling pathway activation and changes in GABAergic interneuron plasticity. Moreover, this study sought to identify whether EA exerts prolonged analgesic and neuroprotective effects following MDPI administration.

## 2 Materials and methods

### 2.1 Animals

Adult male and female ICR mice (four weeks old, 25-30 g) were obtained from BioLasco Taiwan Co., Ltd., Taipei, Taiwan. Five animals were housed per cage under standard laboratory conditions (12:12 light/dark cycle with food and water available *ad libitum*). All necessary efforts were made to ensure the welfare of animals.

### 2.2 Experimental dental pulp injury model

Based on the type of pulpal reaction, DPI models are categorized as follows: type 1, transient injury with no pulp loss; type II: Intermediate injury with peripheral pulp loss; and type III: Irreversible pulpitis characterized by pulp exposure, necrosis, and systemic defense (12). A type III DPI model was selected for the current study. Mice were anesthetized by an intraperitoneal (i.p.) injection of chloral hydrate (0.40 mL/100 g body weight). The animals were initially assigned to three experimental groups: Control, SDPI, and MDPI. In the SDPI group, the dental pulp of the left first maxillary molar was exposed to an air-cooled high-speed dental handpiece and a new #36 round bur. Exposure was confirmed using endodontic K file #6. In the MDPI group, mechanical exposure of the left second maxillary pulp was performed one week after pulp exposure of the left first maxillary molar. The tooth/teeth cavities in the MDPI and SDPI animals remained open for 21 or 70 days. Animals in the control group underwent no dental procedures and only received anesthesia. The mouse’s mouth of each group was held open using a handmade mouth gag placed between the upper and lower left third molars.

### 2.3 Electroacupuncture stimulation procedures

Mice in the EA groups were initially placed in an induction chamber for 15 min, with the oxygen flow meter adjusted to approximately 2.0 L/min (37). For each 20 min acupuncture session, a mask was connected to a non-rebreathing circuit (Bain) with 1.5% isoflurane (Panion & BF Biotech Inc., Taoyuan, Taiwan). A stimulator (Trio-300; Ito, Japan) was applied at an intensity of 150 µs square pulses of 2 mA at 2 Hz. The acupuncture needles (Shanghai Yanglong Medical Articles Co., Ltd., Shanghai, China) differed in size: 36-gauge for the distal acupoints and 32-gauge for sham acupoints.

### 2.4 Distal and sham EA groups

Twelve cumulative EA sessions were conducted for each mouse. Once a day, sessions started one day (1 d) after MDPI and on days two, three, seven, eight, nine, 13, 14, 15, 19, 20, and 21. EA sessions were delivered on the ipsilateral side (left forelimb) of the injured teeth (**Figure S1**). The anatomical location of the acupoints was determined according to a previously described trans-positional acupoint system (38).

The Hegu (LI4) and Quchi (LI11) acupoints served as the distal acupoints. LI4 is located on the first dorsal interossei, on the radial side to the midpoint of the second metacarpal bone of the index finger, whereas LI11 is located in the depression at the lateral end of the transverse cubital crease of the humerus (38).

The pair of sham acupoints were located (4 mm away from each other) in the middle part of the lateral deltoid muscle in an area distant from most meridians, as previously described (39). The selection of appropriate acupoints was based on two of the three principles that practitioners use: distal acupoints located (1) along the meridians and (2) based on symptom differentiation (40).

### 2.5 von Frey testing

The mechanical response threshold of the withdrawal behavior was measured using an electronic von Frey instrument (BIO-EVF4; Bioseb, Vitrolles, France). All behavioral procedures were conducted in a quiet room under dim light conditions. Furthermore, 1 h before testing, the mice were placed in a plastic cage for habituation. The experimenter held the mice gently with minimal restraint, and the tip was presented perpendicular to the surface of the left cheek side (SDPI or MDPI territory) with sufficient force to cause withdrawal, as previously reported (41). The hard tip was applied thrice on the injured side within a 1 min interval between trials for each mouse (42). The responses were considered positive only when a fast withdrawal reaction was observed. The study did not consider negative responses, such as an attempt to attack the stimulation object or unilateral or bilateral facial grooming. Following baseline observations, withdrawal thresholds were considered for evaluations in the SDPI group on days minus three and one and continued one day after the sole pulp exposure (day one) and then on days three, seven, 14, 21, 28, 35, 42, 49, 56, 63, and 70. In the MDPI group, withdrawal thresholds were considered one day after the first pulp exposure (minus three days) and on minus one day, then resumed one day after the second pulp exposure (day one), and then on days three, seven, 14, 21, 28, 35, 42, 49, 56, 63, and 70.

### 2.6 Immunofluorescence

Coronal sections of the brainstem of 30 µm were processed for IF staining as previously reported (43). The sections were suspended and blocked for 60 min with 10% donkey serum in PBS containing 0.4% Triton X-100 before being incubated overnight at 4°C with a primary antibody (mouse anti-GFAP (1:500; Cell Signaling Technology, 3670), rabbit anti-GS (1:1000; Abcam, ab49873)). After three washes with PBS, the sections were incubated with the appropriate secondary antibody for 60 min at room temperature (1:250, Alexa Fluor donkey anti-mouse 488, Abcam, ab150105; 1:250, Alexa Fluor donkey anti-rabbit 594, Abcam, ab1500776), washed, mounted (ProLong™ Diamond Antifade Mountant with DAPI, Thermo Fisher P36971), and coverslipped.

For GAD65 immunostaining, we used a specific protocol with some modifications (44). The brainstem sections were washed in 0.1M Tris (pH7.6), followed by gentle rinsing with 3% H_2_O_2_ before being blocked first with Tris-BSA (0.1M Tris, pH 7.6, 0.05% BSA) for 15 min and then with 10% donkey serum in Tris-BSA for 60 min at room temperature. The sections were incubated overnight at 4°C with primary antibody (mouse anti-GAD65 (1:300; Abcam, ab26113). After three washes with Tris-BSA, the sections were incubated with a secondary antibody for 60 min at room temperature (1:250, Alexa Fluor donkey anti-mouse 488, Abcam, ab150105), washed, and mounted. Fluorescent images were captured using a confocal microscope (Leica SP5 TCS; Heidelberg, Germany).

### 2.7 Western blot

Tissue samples of the Vi/Vc transition zone area of the brainstem were extracted at 21 or 70 days after injury and stored at -80°C until further processing as per previously described procedures (45). Samples of approximately 20 or 30 µg of protein were loaded onto precast 8% or 12% SDS-PAGE gels. The PVDF membranes were blocked with 5% BSA in Tris-buffered saline for 1 h at room temperature and incubated overnight at 4°C with the following primary antibodies: mouse anti-GFAP (1:2000; #3670; CST), rabbit anti-GS (1:15000; ab49873; Abcam), guinea pig Anti-GLT-1 (1:2000; AB1783; Merck Millipore), rabbit anti-Cleaved Caspase-3 (1:2000; Asp175; CST), rabbit anti-Bax (1:2000; #2772; CST), rabbit anti-Bcl-2 (1:2000; GTX100064; CST), and mouse anti-GAD65 (1:2000; ab26113; Abcam). Signals were normalized to those of donkey anti-guinea pig IgG antibody (1:10,000; ab6908; Abcam) and β-actin (1:10,000; GTX629630) as an internal control. Signals were digitally scanned using the FUSION FX software.

### 2.8 Statistical analyses

Results are expressed as the mean ± standard error of the mean (SEM), and *p* values <0.05 were considered statistically significant. For behavioral investigations, between-group comparisons were performed using repeated-measures of analysis of variance (ANOVA), followed by Bonferroni’s *posthoc* test. In addition, relative protein expression levels in different groups were compared using a one-way ANOVA, followed by Dunnett’s T3 *posthoc* test. Data were analyzed using SPSS (v20, IBM, Armonk, NY, USA).

## 3 Results

### 3.1 Behavioral responses of mice following single-tooth DPI or MDPI

**Figure 1:**
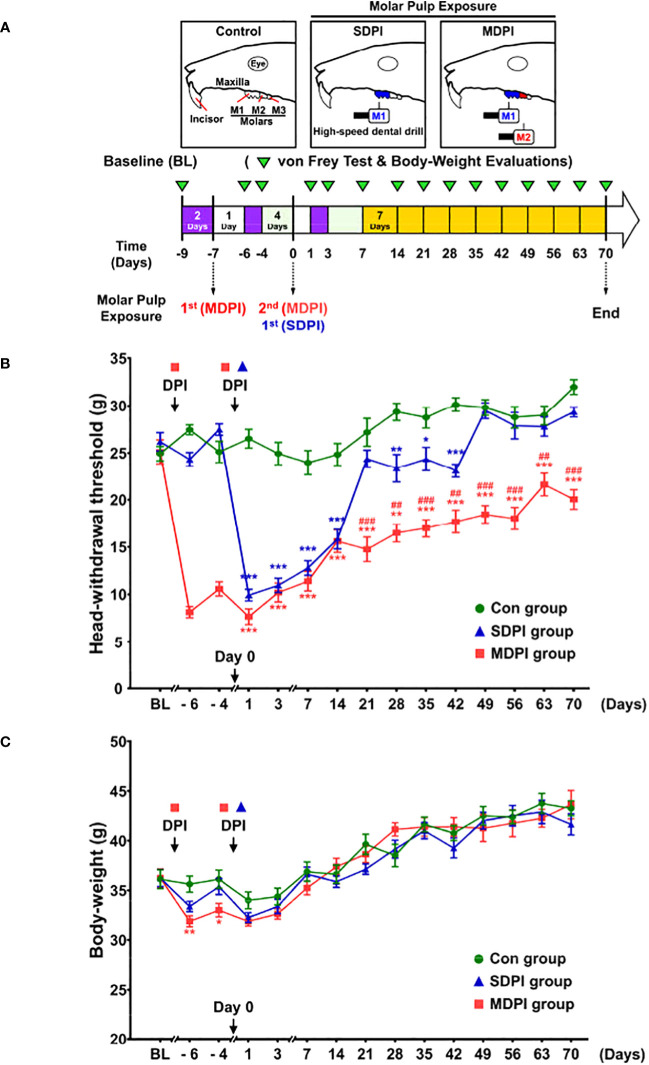
Mechanical nociceptive thresholds and body weight measurements at various time points after SDPI or MDPI **(A)** Schematic presentation of the experimental study design. **(B)** Analysis of the head withdrawal thresholds (HWT) in mice measured by the von Frey test. From days one to 14, SDPI or MDPI mice displayed a significant decrease in HWT compared to the controls. There was no significant difference in HWT at either time point between SDPI or MDPI mice. From days 49 to 70, responses from the SDPI mice were restored to nearly control values, whereas, from days 21 to 70, MDPI mice exhibited a persistent lower HWT compared to the controls or SDPI counterparts. **(C)** Transient effects of MDPI but not SDPI in mice body weight. Data are presented as the mean ± SEM. Between-group comparisons were performed by repeated-measures ANOVA at each time point, followed by Bonferroni’s *posthoc* test (^*^ *p*<0.05, ^**^ *p*<0.01, ^***^ *p*<0.001 vs. control; ^##^ *p*<0.01, ^###^ *p*<0.001, vs. SDPI; *n*=9 in each group).

To closely induce one of the most remarkable clinical features of irreversible pulpitis (e.g., severe pain sensation), we developed a modified version of the existing DPI model. Initially, we compared the duration and severity of the head withdrawal threshold (HWT) between mice subjected to SDPI and our modified MDPI model for >70 days (**Figure 1A**). All experimental groups, including control, SDPI, and MDPI, showed similar responses to the von Frey test at baseline, with a withdrawal threshold of approximately 25 g (**Figure 1B**). Mice that underwent SDPI showed dramatic changes in HWT evaluation from days one to 14 (**Figure 1B**). A return to the control values was displayed by SDPI mice on day 21, followed by a secondary significant decrease in HWT on day 28, which was also noted on days 35 and 42 compared to the control values (**Figure 1B**). From day 49 until the end of our evaluations on day 70, the HWT increased and returned to the control values (**Figure 1B**). In MDPI mice, an initial marked decrease in HWT was also observed from days one to 14 after pulp exposure of the second molar (**Figure 1B**). From days 21 to 70, MDPI mice exhibited a significant and constant decrease in HWT, and this change did not remit to the control values at either time point (**Figure 1B**). A repeated-measures ANOVA determined that the mean HWT were significantly difference across three groups (F (2, 21) = 446.6, p <0.001). Moreover, a *posthoc* pairwise comparison using the Bonferroni correction showed a decreased HWT between the control and SDPI (27.3 vs 22.4, p<0.001), between the control and MDPI (27.3 vs 15.5, p<0.001), between SDPI and MDPI (22.4 vs 15.5, p<0.001) at overall time point. These results suggest that MDPI successfully induced a long-lasting orofacial pain-like sensation that persisted for 70 days.

### 3.2 SDPI or MDPI models induce only temporal changes in the body weight of mice

Next, we evaluated body weight loss in SDPI and MDPI mice. Body weight loss in rodents is considered an objective indicator of decreased appetite due to pain-like sensations (46). To evaluate possible persistent starvation and, thereby, significant weight loss induced by SDPI or MDPI, mice were weighed following the von Frey test schedule (**Figure 1A**). At baseline, all three groups exhibited similar body weights of approximately 36 g (**Figure 1C**). MDPI mice that underwent the first pulp exposure showed a significant decrease in body weight on days minus six and four compared to the controls. There was no significant difference in body weight between the SDPI and control groups (**Figure 1C**). Subsequently, mice that experienced a second pulp exposure (MDPI), sole pulp exposure (SDPI), and control mice exhibited a marked body weight loss on days one and three, and there was no significant difference in body weight between the three groups (**Figure 1C**). From day seven onwards, all three studied groups started to regain weight throughout the end of our evaluations at 70 days, and there was no significant difference between them (**Figure 1C**). A repeated-measures ANOVA determined that the mean body weight was slightly different across three groups (F (2, 21) = 3.7, *p*=0.043). There was no significant difference in body weight between the SDPI and control groups (**Figure 1C**; *p*=0.072 by Bonferroni’s *posthoc* test); and between the MDPI and control groups (**Figure 1C**; *p*=0.106 by Bonferroni’s *posthoc* test) at overall time point. These findings suggest that MDPI may induce only transient weight loss.

### 3.3 Morphological and functional changes of astrocytes in the Vi/Vc transition zone following SDPI or MDPI

Emerging studies suggest that alterations in astrocytic signaling molecules, such as GLT1 and GS, appear to play a crucial role in the pathophysiological mechanisms of pain chronicity, and the significance of these changes is poorly understood (20, 47, 48).

**Figure 2:**
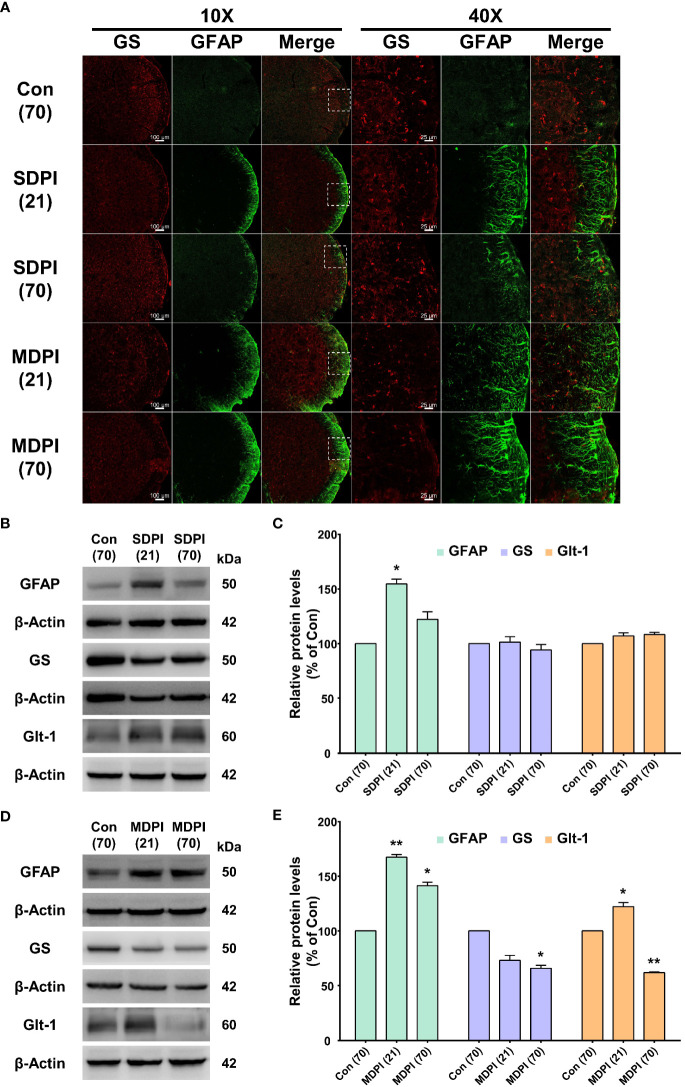
Double immunofluorescence staining and western blot (WB) analysis at 21 and 70 days after SDPI or MDPI. **(A)** Representative confocal microscopy images (10x and 40x) for GFAP (green) and GS (red) in the Vi/Vc of the mice brainstem. Remarkable GFAP immunoreactivity (GFAP-IR) astrocytes were noted in the SDPI-21-day group but almost undetectable in the SDPI-70-day group. No changes were observed in GS immunoreactivity (GS-IR) astrocytes in SDPI-21-day and SDPI70-day groups. Increased and persistent GFAP-IR astrocytes along with a gradual decrease in GS-IR astrocytes in the MDPI-21-day and MDPI-70-day groups; scale bars=100 µm (10x) and 25 µm (40x). **(B–E)** Representative WB bands and histograms, respectively, depict the expression of GFAP, GS, and Glt-1 at 21 and 70 days following SDPI **(B, C)** and MDPI **(D, E)**. Data are presented as the mean ± SEM. Between-group comparisons were performed by a oneway ANOVA, followed by Dunnett’s T3 *posthoc* test (^*^ *p*<0.05, ^**^ *p*<0.01 vs. control; *n*=5 in each group).

To evaluate the morphological and functional changes of astrocytes in the Vi/Vc transition zone of the brainstem following SDPI or MDPI, the expression of glial fibrillary acidic protein (GFAP), GS, and glutamate GLT-1 antibodies was analyzed by immunofluorescence (IF) and western blot (WB). Tissue samples were collected at two-time points: 21 and 70 days after the injury/injuries. We first analyzed IF double staining for GFAP and GS antibodies. Activated astrocytes can be identified by increased GFAP immunoreactivity (GFAP-IR) and exhibit a hypertrophic shape with remarkable GFAP expression in all major branches and fully extended processes (48). In our IF evaluations, astrocytes showed considerable activation in the SDPI21-day group compared to the controls (**Figure 2A**). However, GFAP-IR astrocytes were not remarkable in the SDPI-70-day group compared to the controls (**Figure 2A**). Similarly, strong astrocyte GFAP-IR was observed in the MDPI-21-day group, and such notable immunoreactivity was also present in the MDPI-70-day group compared to the controls (**Figure 2A**). We then evaluated GS immunoreactivity (GS-IR) in astrocytes, a specific enzyme that is mainly detected in the cytoplasm and presents as a discrete non-uniform pleomorphic structure (47). Double IF staining showed no remarkable changes in GS-IR astrocytes in the SDPI-21 and SDPI-70-day groups compared to the controls (**Figure 2A**). However, we noted a trend toward decreased GS-IR astrocytes in the MDPI-21-day and MDPI-70-day groups compared to the controls (**Figure 2A**).

To complement our histological evaluations and to confirm and characterize the extent of astrocyte activation, the levels of GFAP, GS, and GLT-1 proteins were evaluated by WB. The levels of GFAP protein were significantly upregulated in the SDPI-21-day group prior to a return to the control levels observed in the SDPI-70-day group (**Figures 2B, C**). In contrast, the GFAP protein levels were remarkably upregulated in the MDPI-21-day group and remained at a higher level in the MDPI-70-day group than in the controls (**Figures 2D, E**). Moreover, no changes were observed in GS and GLT-1 protein levels in the SPDI-21-day and SDPI-70-day groups compared to the controls (**Figures 2B, C**). Furthermore, the GS protein expression decreased gradually, as observed in the MDPI-21-day group, and was significantly downregulated in the MDPI-70-day group compared to the controls (**Figures 2D, E**). In contrast, GLT-1 protein expression was significantly upregulated in the MDPI-21-day group and downregulated in the MDPI-70-day group compared to the controls (**Figures 2D, E**). Taken together, these results indicate that MDPI induces persistent astrocyte reactivity in the Vi/Vc transition zone involving GLT-1 biphasic changes, along with a gradual downregulation of GS.

### 3.4 Apoptotic activity in the Vi/Vc transition zone after peripheral nerve injury induced by SDPI or MDPI

**Figure 3:**
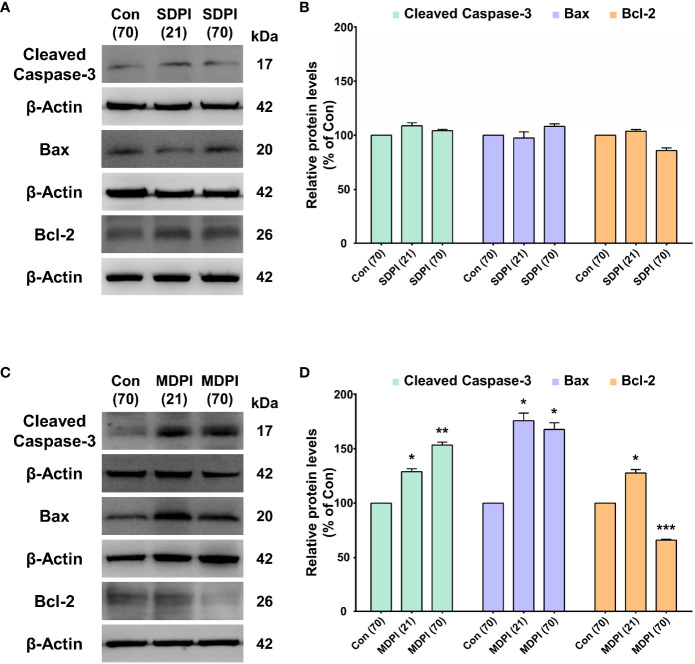
Assessment of apoptosis-related protein expression in the Vi/Vc after SDPI or MDPI. **(A-D)** Immunoblotting bands and histograms, respectively, depicting the levels of cleaved caspase-3, pro-apoptotic protein Bax and anti-apoptotic protein Bcl-2, and β-actin as a loading control at 21 and 70 days following SDPI or MDPI, respectively. **(A, B)** The cleaved caspase3, Bax, and Bcl-2 protein levels remained constant and comparable to the control levels in the SDPI-21 and SDPI-70-day groups. No significant differences were observed between groups. **(C, D)** Progressive upregulation of cleaved caspase-3 or Bax proteins in the MDPI-21-day and MDPI-70-day groups compared to the controls. Significant upregulation of Bcl-2 protein was detected in the MDPI-21-day group, whereas a significant downregulation was observed in the MDPI-70-day group compared to the controls. Data are presented as the mean ± SEM. Betweengroup comparisons were performed by a one-way ANOVA, followed by Dunnett’s T3 *posthoc* test (^*^ *p*<0.05, ^**^ *p*<0.01, ^***^ *p*<0.001 vs. control; *n*=5 in each group).

To evaluate the involvement of apoptotic signaling molecules in the nociceptive pathways induced by SDPI or MDPI, we investigated the levels of cleaved caspase-3, Bax, and Bcl-2 proteins in Vi/Vc. Our WB analysis showed no changes in the cleaved caspase-3 protein levels in the SDPI-21-day and SDPI-70-day groups compared to the controls (**Figures 3A, B**). However, there was a significant and progressive upregulation in the cleaved caspase-3 protein levels between the MDPI-21-day and MDPI-70-day groups compared to the controls (**Figures 3C, D**). Moreover, the Bax and Bcl-2 protein levels in both SDPI-21-day and SDPI-70-day groups remained unchanged compared to the controls (**Figures 3A, B**). However, there was a significant upregulation of the Bax protein levels in the MDPI-21-day group, which remained significantly higher in the MDPI-70-day group than in the controls (**Figures 3C, D**). In contrast, the Bcl-2 protein levels significantly increased in the MDPI-21-day group, while there was a remarkable downregulation in the Bcl-2 protein levels in the MDPI-70-day group compared to the controls (**Figures 3C, D**). These findings suggest that the pro-apoptotic proteins, such as cleaved caspase-3 and Bax, and the anti-apoptotic protein Bcl-2 might be associated with persistent pain induced by MDPI.

### 3.5 GAD65 expression of GABAergic interneurons in the Vi/Vc transition zone following SDPI or MDPI

**Figure 4:**
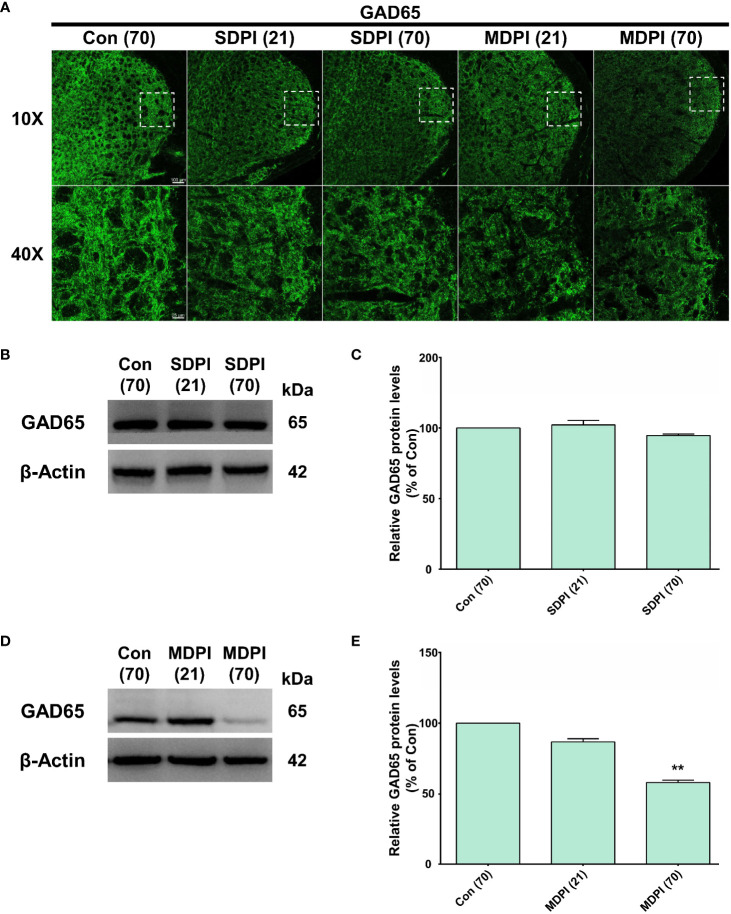
Evaluation of GAD65 expression in the Vi/Vc transition zone following SDPI or MDPI. **(A)** Representative confocal microscopy images of GAD65 immunoreactivity (GAD65-IR) in the Vi/Vc at 21 and 70 days following SDPI or MDPI. There was a strong presence of GAD65IR in three experimental groups: SDPI-21-day, SDPI-70-day, and controls, whereas decreased GAD65-IR was observed in the MDPI-70-day group. Magnified views of the brainstem (10x and 40x); scale bars=100 µm (10x) and 25 µm (40x). **(B–E)** Representative WB bands and histograms depicting changes in the expression of GAD65 protein after SDPI **(B, C)** or MDPI **(D, E)**. There was a significant decrease in GAD65 in the MDPI-70-day group compared with the control group. Data are presented as the mean ± SEM. Between-group comparisons were performed by a one-way ANOVA, followed by Dunnett’s T3 *posthoc* test (^**^ *p*<0.01 vs. control; *n*=5 in each group).

IF and WB assessments were performed to analyze the role of the GAD65 isoform of GABAergic interneurons in orofacial pain induced by SDPI or MDPI. In our IF evaluations, GAD65-immunoreactivity (GAD65-IR) was identified as numerous punctate structures of variable size, consistent with previous literature (49). There was a remarkable amount of GAD65-IR well-distributed throughout the entire Vi/Vc transition zone in the SDPI-21-day and SDPI-70-day groups, as well as in the controls (**Figure 4A**). In contrast, we noted a trend toward decreased GAD65-IR between the MDPI-21-day and MDPI-70-day groups compared to the controls (**Figure 4A**). Consistent with our IF evaluations, our WB analysis demonstrated no significant changes in the GAD65 protein levels in the SDPI-21-day and SDPI-70-day groups compared to the controls (**Figures 4B, C**). However, the GAD65 protein levels decreased, as observed in the MDPI-21-day group, and were significantly downregulated in the MDPI-70day group compared to the controls (**Figures 4D, E**). Overall, these results suggest that the downregulation of GAD65 expression is associated with MDPI.

### 3.6 Duration of the analgesic effect of cumulative EA treatment in an MDPI model

**Figure 5:**
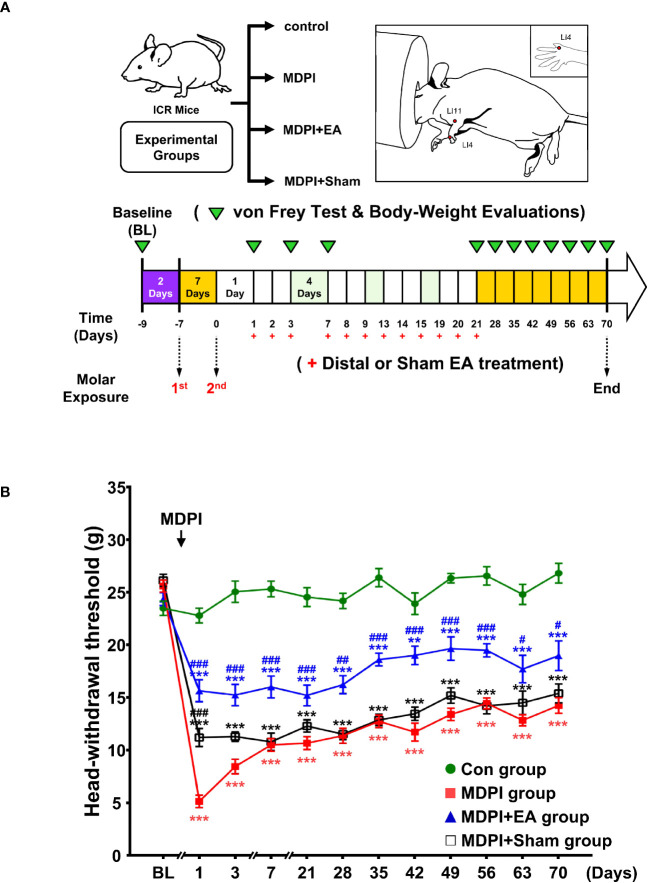
Mechanical allodynia using von Frey stimulation after distal or sham EA treatment in MDPI mice. **(A)** Schematic diagram of the experimental study design. **(B)** Baseline of the HWT to von Frey stimulation in the MDPI, MDPI+EA, MDPI+sham, and controls groups. Effect of EA or sham EA treatment on the HWT from days one to 21 after MDPI. There were notable signs of decreased HWT in the MDPI group compared to the controls from days one to 21, whereas MDPI+EA significantly attenuated such effects, indicating an analgesic effect. There were no major HWT differences between the MDPI+Sham and MDPI groups during the observation period. **(B)** Continuous evaluation of HWT for a long-term period of 49 days after the cessation of EA or sham EA treatment. The MDPI group exhibited a decreased HWT compared to the controls from days 28 to 70. A significant difference in the HWT between the MDPI+EA and MDPI groups indicated a prolonged analgesic effect on multiple time points after treatment (from days 28 to 70). The MDPI+Sham group showed no significant or long-lasting effects in the HWT during the entire testing period. Data are presented as the mean ± SEM. Between-group comparisons performed by repeated-measures ANOVA at each time point, followed by Bonferroni’s *posthoc* test (^**^ *p*<0.01, ^***^ *p*<0.001 vs. control; ^#^ *p*<0.05, ^##^ *p*<0.01, ^###^ *p*<0.001 vs. MDPI; *n*=9 in each group).

The results presented in **Figure 1** suggest that persistent pain was induced by MDPI but not SDPI. Moreover, considering that interest in the use of acupuncture for pain control in dentistry has increased in recent years (35), we explored the analgesic effects of cumulative EA treatment in MDPI animals. Accordingly, cumulative EA treatment and behavioral assessments were performed (from days one to 21) after MDPI (**Figure 5A**). Baseline responses in all four study groups, including the MDPI, MDPI+EA, MDPI+sham, and control groups, showed an HWT of approximately 25 g (**Figure 5B**). From days one to 21, the MDPI group exhibited a persistent decrease in HWT compared to the controls (**Figure 5B**), consistent with the data depicted in **Figure 1**. Likewise, from days one to 21, the MDPI+EA group showed a significantly increased HWT compared to the MDPI group (**Figure 5B**). Furthermore, the MDPI+Sham group exhibited variable HWT (**Figure 5B**). An initial significantly higher HWT was observed on day one compared to that in MDPI mice (**Figure 5B**). The HWT decreased from days three to 21, and there was no significant difference compared to the MDPI group (**Figure 5B**).

After the endpoint of the EA and sham treatments, HWT was continuously evaluated once a week in all four study groups for a long-term period of 49 days (**Figure 5A**). Therefore, consistent with the data presented in **Figure 1**, the MDPI group showed a significant and persistent decrease in HWT from days 28 to 70 compared to the controls (**Figure 5B**). Moreover, HWT in the MDPI+EA group remained higher than that in the MDPI group multiple days after the endpoint of the treatment, as exhibited on days 28, 35, 42, 49, 56, 63, and 70 (**Figure 5B**). Furthermore, during the second part of the experimental period, there was no significant difference in the HWT between MDPI+ Sham and MDPI groups (**Figure 5B**). A repeated-measures ANOVA determined that the mean HWT was significantly different across three groups (F (3, 32) = 719.3, *p* <0.001). A *posthoc* pairwise comparison using the Bonferroni correction showed a decreased HWT between the control and MDPI (25.0 vs 12.6, *p*<0.001); an increased HWT between the MDPI and MDPI+EA (12.6 vs 18.0, *p*<0.001); and a slightly increased HWT between the MDPI and MDPI+sham (12.6 vs 14.1, *p*<0.001) at overall time point. These results suggest that 12 sessions of cumulative EA treatment at distal acupoints provide a long-lasting analgesic effect for at least 49 days after the cessation of EA treatment in an MDPI model.

### 3.7 Cumulative EA treatment exerts prolonged astrocyte inhibition involving GS and GLT-1 modulation

**Figure 6:**
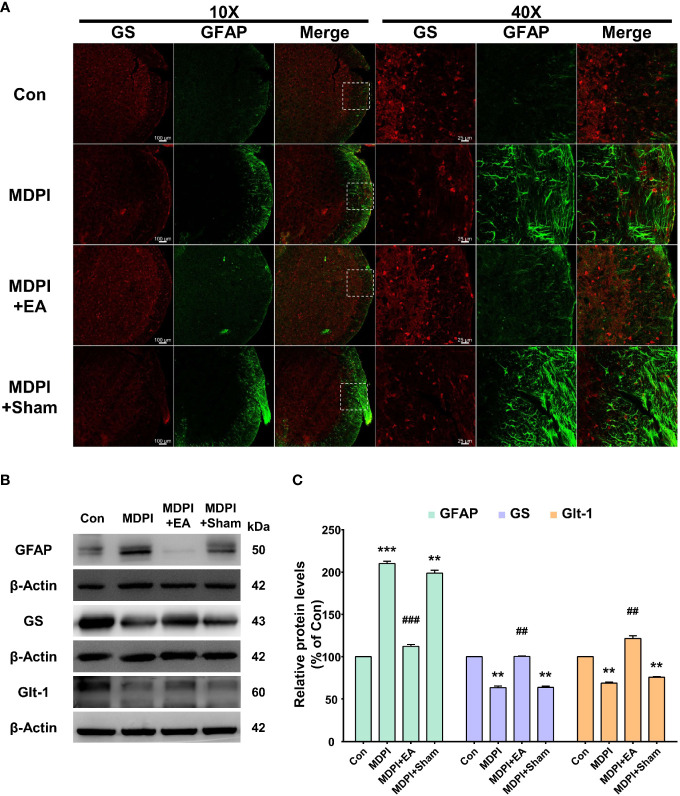
Prolonged effects of cumulative EA treatment in astrocytes activity involving GS or GLT-1 modulation. **(A)** Double immunoreactivity GFAP and GS were analyzed in the Vi/Vc transition zone. Increased GFAP-IR was noted in the MDPI-70-day group. EA treatment resulted in a longlasting inhibition of GFAP-IR observed 49 days after the cessation of treatment (day 70 after MDPI). The MDPI+Sham-70-day group exhibited a similar GFAP-IR pattern to the MDPI-70day group. GFAP colocalized with GS showing lower GS-IR in the MDPI-70-day and MDPI+Sham-70-day groups. There was increased GS-IR in the MDPI+EA-70-day group. Magnified views of the brainstem (10x and 40x); scale bars=100 µm (10x) and 25 µm (40x). **(B, C)** Representative WB bands and histogram, respectively, depict the expression of GFAP, GS, Glt-1, and β-actin as a loading control. Data are presented as the mean ± SEM. Betweengroup comparisons were performed by a one-way ANOVA, followed by Dunnett’s T3 *posthoc* test (^**^ *p*<0.01, ^***^ *p*<0.001 vs. control; ^##^ *p*<0.01, ^###^ *p*<0.001 vs. MDPI-70-day; *n*=5 in each group).

To better evaluate the long-lasting duration of the analgesic effect of cumulative EA and sham treatment, we analyzed changes in GFAP, GS, and GLT-1 expression in Vi/Vc samples collected on the last day of our evaluation at day 70 (49 days after the end of EA treatment). Double IF for GFAP and GS antibodies was consistent with the results shown in **Figure 2**. The MDPI-70-day group exhibited an intense GFAP-IR compared to the controls (**Figure 6A**). In contrast, there was low GFAP-IR in the MDPI+EA-70-day group compared to the MDPI-70day group (**Figure 6A**). However, in the MDPI+Sham-70-day group, similar GFAP-IR was observed compared to the MDPI-70-day group (**Figure 6A**). Furthermore, there was low GS-IR in the MDPI-70-day group compared to the controls (**Figure 6A**). However, GS-IR was increased in the MDPI+EA-70-day group compared to the MDPI-70-day group (**Figure 6A**). The GS-IR in the MDPI+Sham-70-day group resembled the GS-IR in the MDPI-70-day group (**Figure 6A**).

Additionally, we performed WB to examine GFAP, GS, and GLT-1 protein expression. The GFAP protein levels were significantly upregulated in the MDPI-70-day group compared to the controls (**Figures 6B, C**). In contrast, the GFAP protein levels were remarkably downregulated in the MDPI+EA-70-day group compared to the MDPI-70-day group (**Figures 6B, C**). However, there was no significant difference in the GFAP protein levels between the MDPI+Sham-70 and MDPI-70-day groups (**Figures 6B, C**). Moreover, the GS and GLT-1 protein levels were significantly decreased in the MDPI-70-day group compared to the controls (**Figures 6B, C**). However, the GS and GLT-1 protein levels were notably increased in the MDPI+EA70-day group compared to the MDPI-70-day group (**Figures 6B, C**). There was no significant difference in the GS and GLT-1 protein levels between the MDPI+Sham-70-day and MDPI70-day groups (**Figures 6B, C**).

Overall, these results suggest that cumulative distal EA treatment may be associated with prolonged inhibition of astrocyte activation and modulation of the astrocytic GS and GLT-1 levels following MDPI.

### 3.8 Long-lasting regulation of apoptotic markers in the MDPI model following cumulative EA treatment

To implicate a prolonged modulation of apoptosis signaling pathways with cumulative EA or sham treatment in an MDPI model, the apoptosis-related proteins (cleaved caspase-3, Bax, and Bcl-2) were examined by WB in mice Vi/Vc samples collected at 70 days.

**Figure 7:**
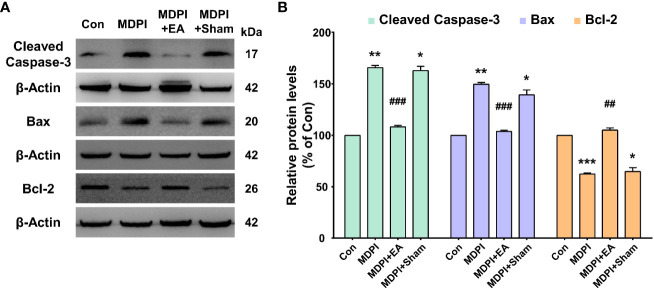
Prolonged modulatory effects of cumulative EA treatment on apoptotic markers in the MDPI model. **(A, B)** Representative WB bands and histograms, respectively, depict the levels of cleaved caspase-3, Bax and Bcl-2, and β-actin as a loading control. Significant upregulation of the cleaved caspase-3 and Bax protein levels was detected in the MDPI-70-day and MDPI+Sham70-day groups but not in the MDPI+EA-70-day group compared to the controls. The cleaved caspase-3 and Bax protein levels were significantly downregulated in the MDPI+EA-70-day group compared to the MDPI-70-day group but not in the MDPI+Sham-70-day group. The Bcl-2 protein levels were significantly downregulated in the MDPI-70-day and MDPI+Sham70-day groups but not in the MDPI+EA-70-day group compared to the controls. Data are presented as the mean ± SEM. Between-group comparisons were performed by a one-way ANOVA, followed by Dunnett’s T3 *posthoc* test (^*^ *p*<0.05, ^**^ *p*<0.01, ^***^ *p*<0.001 vs. control; ^##^ *p*<0.01, ^###^ *p*<0.001 vs. MDPI-70-day; *n*=5 in each group).

The levels of cleaved caspase-3 and Bax proteins were significantly upregulated in the MDPI70-day group compared to the controls (**Figures 7A, B**). In contrast, the cleaved caspase-3 and Bax protein levels were significantly downregulated in the MDPI+EA-70-day group compared to the MDPI-70-day group (**Figures 7A, B**). Moreover, there was no significant difference in the cleaved caspase-3 and Bax protein levels between the MDPI+Sham-70-day and MDPI-70-day groups (**Figures 7A, B**). Furthermore, the Bcl-2 protein levels were significantly downregulated in the MDPI-70-day group compared to the controls (**Figures 7A, B**). The levels of Bcl-2 protein were notably upregulated in the MDPI+EA-70-day group compared to the MDPI-70-day group (**Figures 7A, B**). However, there was no significant difference in the Bcl-2 protein levels between the MDPI+Sham-70-day and MDPI-70-day groups (**Figures 7A, B**).

### 3.9 Prolonged effects of cumulative EA treatment in GAD65 expression of GABAergic interneurons in an MDPI mice model

**Figure 8:**
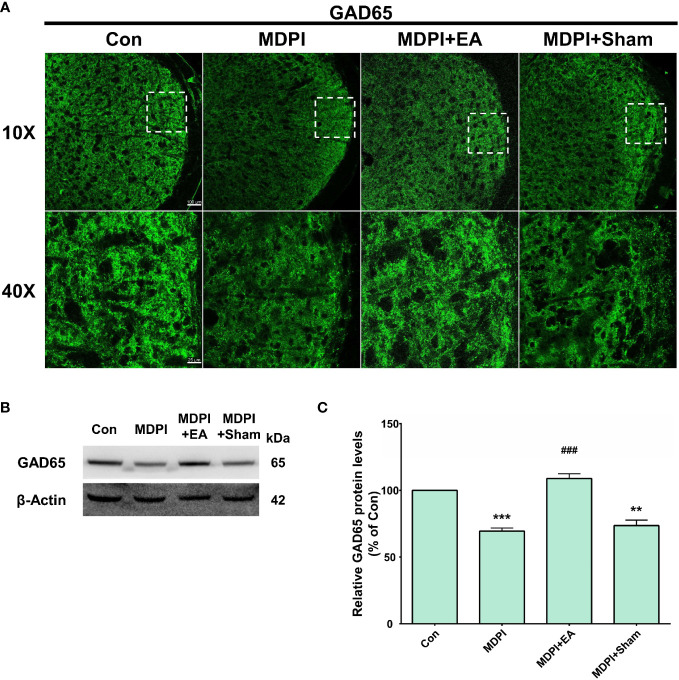
Prolonged effects of cumulative EA treatment in GAD65 expression in the MDPI model. **(A)** Representative confocal images (10x and 40x) of GAD65-IR in the Vi/Vc transition zone of the four groups: control, MDPI-70-day, MDPI+EA-70-day, and MDPI+Sham-70-day. GAD65-IR was markedly reduced in the MDPI-70-day group, whereas the MDPI+EA-70-day group exhibited high GAD65-IR. The MDPI+Sham-70-day and MDPI-70-day groups exhibited similar GAD65-IR; scale bars=100 µm (10x) and 25 µm (40x). **(B, C)** Illustrative WB bands and histogram, respectively, depicting GAD65 protein expression and β-actin as a loading control. Data are presented as the mean ± SEM. Between-group comparisons were performed by a one-way ANOVA, followed by Dunnett’s T3 *posthoc* test (^**^ *p*<0.01, ^***^ *p*<0.001 vs. control; **^###^***p*<0.001 vs. MDPI-70-day; *n*=5 in each group). .

IF and WB analyses were performed to explore the prolonged effect of cumulative EA and sham treatment on GAD65 expression in the MDPI model. Mouse tissue samples were collected following previously reported methods, as reported in **Figure 6**. IF evaluations revealed a remarkable amount of GAD65-IR in the controls, while there was lower GAD65-IR in the MDPI-70-day group, consistent with the data presented in **Figure 4** (**Figure 8A**). In contrast, there was intense GAD65-IR in the MDPI+EA-70-day group, whereas the MDPI+sham-70day group exhibited decreased GAD65-IR (**Figure 8A**). Additionally, there was a similar intensity of GAD65-IR between the MDPI+Sham-70-day and MDPI-70-day groups (**Figure 8A**). In accordance with our IF evaluations, WB analysis demonstrated significant downregulation of the GAD65 protein levels in the MDPI-70-day group compared to the controls (**Figures 8B, C**). Moreover, the GAD65 protein levels were significantly upregulated in the MDPI+EA-70-day group compared to the MDPI-70-day group (**Figures 8B, C**). There was no significant difference between the MDPI+sham-70-day and MDPI-70-day group (**Figures 8B, C**).

## 4 Discussion

To the best of our knowledge, this is the first study to describe the establishment of a reliable persistent orofacial pain model induced by MDPI. In line with this conclusion, we have provided experimental evidence of the various mechanisms involved in trigeminal central sensitization, focusing on astrocyte reactivity, activation of apoptotic signaling pathways, and GABAergic interneuron plasticity. Moreover, this study also documented for the first time the prolonged analgesic effects of EA alleviating mechanical allodynia induced by MDPI for over 49 days after the end of treatment. Furthermore, in association with the analgesic effects, we also reported that EA exerts the long-term modulation of the central mechanism involved in persistent dental pain induced by MDPI.

### 4.1 Multiple dental pulp injury model: a persistent orofacial pain model

The DPI type III experimental model has been widely used in pain research in dentistry (11). This model varies in terms of the animal utilized, the site of pulp exposure, and the number of teeth injured. Regardless of the approach chosen by the investigator, only short-term anxiety and pain-like behaviors have been commonly reported in animals that underwent dental injury/injuries in a single-day procedure (5, 6, 13, 14, 50). However, a study by Tsuboi et al. described nociceptive responses of approximately 28 days in rats subjected to pulp exposure of the first mandibular molar (15). In agreement with these reports, we observed an initial dramatic nociceptive response to mechanical stimuli on days one, three, and seven, followed by a progressive recovery phase that exhibited complete alleviation of pain-like behavior between days 49 and 70 in the SDPI animals in the current study (**Figure 1B**).

Moreover, increasing scientific evidence suggests that repetitive noxious stimuli can trigger prolonged nociceptive responses in rodents (51–53). In addition, acute pain induced by mechanical or thermal injury can progress to chronic pain when repeated noxious stimuli persist (54). Similarly, we observed persistent nociceptive responses to mechanical stimulation (mechanical allodynia) in MDPI animals as a result of pulp exposure of the first and second maxillary molars, with an interval of seven days between injuries (**Figure 1B**). Our results are also in agreement with previous research involving modifications of the Sluka animal model, which described chronic hyperalgesia induced by repeated and spaced injections of acidic saline in the hind paws of mice (53).

In addition, the time course and intensity of the nociceptive response in MDPI animals from days one to 14 post-injury were comparable to the decreased nociceptive response in SDPI mice, suggesting that there is a similarity in the onset of pain-like behavior between the two groups (**Figure 1B**). However, the nociceptive response was shorter in SDPI mice than in MDPI mice. These findings suggest that the initiation phase of persistent orofacial pain is independent of the extent of dental pulps injured, thereby to the maxillary nerve. In contrast, the time course of the nociceptive response, as reflected in the prolongation of orofacial pain, appears to be related to sustained noxious stimulation of the maxillary nerve. This observation is in agreement with previous research (55).

Regarding a possible sex-related differences in pain-like responses induced by SDPI or MDPI, previous studies have reported similar short-term nociceptive behaviors between male and female mice following a unilateral or bilateral pulp injury/injuries (5, 13). However, increasing scientific evidence suggests that orofacial pain conditions in rodents induce different behavioral responses between the sexes (56, 57). In agreement with these studies, in a pilot study, we observed a persistent nociceptive response in female mice following SDPI or MDPI (**Supplemental Figure 1A**). However, MDPI but not SDPI induces persistent pain-like responses in male mice (**Figure 1B**). Therefore, we assume that pain-like responses may be sex dependent following a single dental pulp injury. However, further studies are needed to explore the differences between the sexes in acute and chronic orofacial pain conditions.

Furthermore, in the present study, we have reported significant short-term decreases in total body weight at the initial stages in MDPI animals but not in SDPI mice (**Figure 1C**), and this finding is consistent with previous related work (13). Likewise, MDPI and SDPI mice regained an equivalent amount of weight as control animals throughout the 70 days observation period (**Figure 1C**). This result suggests that neither MDPI nor SDPI significantly affected the body weight of mice.

This novel MDPI model better mimics the duration of a clinical toothache induced by an irreversible pulpitis profile. However, more evidence is needed to better understand the peripheral and central neuronal circuits associated with different states of dental pain.

### 4.2 Morphological and functional changes of astrocytes in acute and persistent orofacial pain

It has been well documented that the noxious inputs from the oral cavity are processed by the trigeminal sensory nucleus within the brainstem (58). Notably, the maxillary and mandibular tooth pulp afferents are projected to the Vc, Vi/Vc transition zone, Vi, C1/C2, and Pa5 areas of the trigeminal nucleus (59–61). However, recent studies have suggested that the Vi/Vc transition zone plays an important role in orofacial deep-tissue pain processing (62, 63). Moreover, it has been reported that morphological and functional changes of glial cells in response to injury contribute to the pain processing pathways of acute and chronic orofacial conditions (64). With regard to the morphological changes following DPI, previous rodent studies involving unilateral injury of the maxillary or mandibular first molar have reported glial activation in the Vc area at an early period within the first 14 days and at a later period up to 28 days (15, 65, 66). Likewise, the same-day injury of the left first and second maxillary molars in mice induced an early significant astrocyte activation in the Vc and Vi/Vc transition zones (6). Similarly, in this study, we observed astrocyte activation in the Vi/Vc 21 days after SDPI that returned to basal levels on day 70, which correlated closely with the progressive attenuation of nociceptive responses (**Figures 2A–C**). Moreover, prolonged astrocyte activation was also observed in the Vi/Vc transition zone following MDPI, accompanied by a parallel persistent nociceptive behavior that lasted for at least 70 days (**Figures 2A, C, D**). These results are in accordance with the time course of glial activation in the Vc and Vi areas (20, 30, or 60 days) previously reported in the infraorbital nerve transection model (ION) (67). In this study, astrocyte activation was observed for the first time and for the longest time period following repetitive orofacial neuropathic injury.

One of the astrocytes’ fundamental functions is the regulation of glutamate homeostasis. It is widely accepted that activated astrocytes lose their capacity to uptake glutamate, which is released by neurons and other astrocytes, during the glutamate-glutamine cycle (68). Such astroglial uptake is mainly mediated by the glutamate transporter GLT-1 and glutamatemetabolizing enzyme GS (68). Therefore, significant impairment in astrocyte function may lead to glutamate-mediated excitotoxicity, which has a detrimental effect on neurons (68). Notably, mounting evidence suggests that changes in the glutamine-glutamate cycle are critical in the induction and maintenance of neuropathic pain chronicity (69). Interestingly, in rats pretreated with complete Freund’s adjuvant (CFA) or interleukin (IL)-1β, the blockade of glutamate transporters (GLT-1, glutamate aspartate transporter (GLAST), and EAAC1), and GS by an intracisternal injection of DL-threo-β-benzyloxyaspartate (TBOA) or methionine sulfoximine (MSO), respectively, suggested that the glutamate-glutamine cycle in astrocytes plays an important role in the initiation of chronic but not acute orofacial pain conditions (70). Consistent with this study, the glutamate-glutamine cycle was not disturbed at any time after SDPI-induced acute pain (**Figures 2A–C**). Additionally, astrocyte activation was observed only at 21 days, accompanied by no changes in the GLT-1 or GS levels at either time point following SDPI (**Figures 2A–C**). It should be noted that although astrocytes contain abundant GLT-1 and GS, and GS activity has been shown to be a useful marker of astrocytes (71), a previous study suggested that GS inhibition by MSO did not affect astrocyte activation, followed by the application of capsaicin to the dental pulp of rats along with daily masseter muscle contraction for over 14 days (72). Moreover, several lines of evidence suggest that GLT-1 expression does not reflect the levels of GFAP (20, 73).

Furthermore, we observed progressive GS downregulation and a parallel initial GLT-1 upregulation that transitioned to a later significant downregulation following MDPI-induced persistent pain (**Figures 2A, C, D**). These results are consistent with the report mentioned above (70) and further supported by *in vivo* and *in vitro* studies that suggest that GS downregulation facilitates astrocytes migration following spinal cord injury (SCI) (74). Additionally, accumulating evidence suggests that SCI induces astroglial GLT-1 downregulation in the spinal dorsal horn and significant upregulation in the supraspinal brain regions that are responsible for pain processing pathways (i.e., the periaqueductal gray (PAG), thalamus, and amygdala) (75, 76). Likewise, our results are further supported by a CFA pain model that observed an initial GLT-1 upregulation that progressed to long-lasting downregulation in the PAG (20). We hypothesized that in response to SDPI, astrocytes underwent a mild to moderate activation that included changes in their morphology as reflected in the upregulation of GFAP expression, and because it was followed by a progressive downregulation of GFAP expression, we assume that this process may induce different gene changes (i.e., cytokines) that exert beneficial properties that facilitate their recovery. Although GFAP expression did not affect astrocyte function or underlying heterogeneity (A1 or A2) (17), there were no changes in GLT-1 and GS following SDPI (**Figures 2A–C**). Therefore, the SDPI did not disrupt glutamate homeostasis. Moreover, astrocyte activation is a continuation of morphological and molecular changes and, in severe cases, induces their proliferation and scar formation, which leads to detrimental effects in the CNS (77). Similarly, in this study, MDPI-induced prolonged morphological changes in astrocytes, along with sustained changes in the key players of glutamate homeostasis (**Figures 2A, C, D**). Therefore, we assumed that MDPI may induce detrimental effects to a certain extent.

Taken together, these results suggest that changes in astrocytic GLT-1 and GS in the Vi/Vc transition zone depend on injury input. Therefore, persistent orofacial inputs induced by MDPI may be associated with prolonged alterations in the glutamate-glutamine cycle.

### 4.3 Involvement of the apoptosis signaling pathway in the development of persistent orofacial pain

The inability to maintain homeostasis between cell proliferation and apoptosis due to injury or infection has been associated with cancer and autoimmune and neurodegenerative diseases, such as Alzheimer’s disease and Parkinson’s disease (78). However, emerging studies suggest the activation of multiple apoptosis-related genes in the initiation and maintenance of peripheral and central nociceptive sensitization after nerve injury (79, 80). Notably, the apoptotic process is mainly regulated by two large families of proteins: the Bcl-2 and caspase families (79). Interestingly, the overexpression of the apoptotic signaling molecules, proapoptotic (caspase-3 or Bax) and anti-apoptotic (Bcl-2), was reported in a streptozotocin (STZ)-induced diabetic peripheral neuropathy of the sciatic nerve (SN) model (81). In accordance with a previous study, we observed the upregulation of cleaved caspase-3, Bax, and Bcl-2 in the Vi/Vc transition zone at 21 days following MDPI-induced persistent pain (**Figures 3C, D**). Therefore, these data suggest the involvement of apoptotic signaling proteins in the development of persistent orofacial pain, which is in agreement with previous reports (82, 83). Moreover, to further support our results, a previous study suggested the initial role of apoptosis signaling pathways in the generation of pain-like behaviors in animals before apoptotic cell death is apparent (27). Additionally, an imbalance between pro- and anti-apoptotic proteins has been associated with cell vulnerability to apoptosis in chronic constriction injury in the SN model (84). In agreement with a previous study, we observed the upregulation of cleaved caspase-3 or Bax and downregulation of Bcl-2 proteins in the Vi/Vc transition zone, accompanied by a prolonged nociceptive response in the MDPI-70-day group (**Figures 3C, D**). In this model, there is a prolonged induction of apoptotic signaling pathways (several weeks), and it has been reported that apoptotic events progress slowly (85). We hypothesized that neuronal or astrocyte death in the Vi/Vc transition zone is associated with a later phase of MDPI-induced persistent orofacial pain. However, this assumption needs further clarification to determine the precise cell type that undergoes an apoptotic process, and the aim of this study was to describe a basic characterization of the mechanisms involved in persistent orofacial pain pathways. Lastly, the association of apoptosis signaling proteins in orofacial pain pathways may depend on the severity and recurrence of noxius stimuli, as we did not observe any pro- or antiapoptotic protein changes in the Vi/Vc transition zone following SDPI-induced acute pain. This assumption is in agreement with that of a previous study (86).

### 4.4 Downregulation of GAD65 expression in GABAergic interneurons after MDPI

Among the multiple pathways involved in the integration of sensory pain information in the CNS, dysfunction between GABAergic and glycinergic inhibition plays an important role in pain mechanisms (30, 87). However, there is considerable controversy regarding the different pathways that may contribute to this altered neurotransmission. Regarding GABAergic inhibitory synaptic transmission, studies have reported that a decrease in the number of GABAergic interneurons, downregulation of either isoform (GAD65 or GAD67), downregulation of GABA transporters, and changes in NKCC1 or KCC2 expression may be involved in disinhibitory mechanisms that result in increased pain sensation (30, 88). Interestingly, immunohistochemical studies showed decreased GAD65 or GAD67 in the medullary dorsal horn (MDH) following chronic constriction injury (CCI) in the ION rat model (89, 90). Similarly, in our study, we observed a slight downregulation of GAD65 in the Vi/Vc transition zone at 21 days in MDPI mice, which reached significance at 70 days in parallel with the presence of persistent pain-like behavior (**Figures 4A, D, E**). Likewise, and to further support our results, an inferior alveolar nerve transection (IANX) rat model has reported a reduced number of GABAergic neurons in the Vc at an early period and morphological and functional GABAergic neuronal changes at a later period in association with extraterritorial neuropathic pain mechanisms (91). Furthermore, other studies suggest that the loss of GABAergic interneurons after ION parallels the presence of apoptotic markers (92). In this study, there was an apparent coincidence between the downregulation of the GAD65 isoform, the presence of cleaved caspase-3, the imbalance of Bax and Bcl-2 ratio, and ongoing pain sensation at 70 days following MDPI (**Figures 3C, D**). Because similar changes did not occur in the early phase, at 21 days, we hypothesized that the imbalance between pro- and anti-apoptotic markers following MDPI may induce an apoptotic process of GABAergic interneurons in the vi/vc transition zone; however, this assumption remains to be elucidated. However, it is also highly likely that GAD65 protein decrement not only indicates GABAergic neuron cell death after nerve injury (85, 93) but may instead reflect a reduced selective regulatory transcription/translation of the GAD65 isoform (93). Moreover, the GAD65 protein levels may be downregulated by other mechanisms that contribute to the induction and maintenance of chronic pain independent of cell death (94, 95). Although these data are inconclusive and require further clarification, our results support the hypothesis of the involvement of central disinhibitory mechanisms associated with the downregulation of GAD65 expression during the maintenance phase of persistent orofacial pain after MDPI.

### 4.5 Prolonged analgesic and neuroprotective effects of cumulative EA following MDPI

In recent years, acupuncture effects have been classified as either immediate (evaluations assessed after the end of the first session) or cumulative effects of multiple acupuncture sessions (96). Currently, most animal models, clinical trials, and systematic reviews have focused on the analgesic effects of cumulative acupuncture in acute and chronic pain conditions (97, 98). However, there is limited experimental evidence of the analgesic effect of cumulative acupuncture treatment in dentistry.

Nevertheless, a mouse model of trigeminal neuralgia suggested that cumulative EA treatment alleviates orofacial allodynia and anxiety-like behaviors induced by infraorbital nerve transection injury (pT-ION) (99). In addition, our previous study demonstrated the analgesic effect of 12 cumulative EA sessions after SDPI (16). Similarly, in this study, we observed that cumulative EA treatment alleviated orofacial mechanical allodynia for 21 days following MDPI (**Figure 5B**). Interestingly, our data also indicate a significant analgesic effect of sham EA treatment, which was observed only on the first treatment day (**Figure 5B**). While this finding seems inconsistent with the inability of Sham acupuncture to alleviate orofacial mechanical allodynia previously reported (16, 99), it is important to note that the general conclusion from meta-analytic approaches is that a minimal analgesic effect of sham acupuncture exists but might lack of clinical relevance (100).

Furthermore, cumulative EA prolonged the alleviation of orofacial mechanical allodynia in MDPI mice for at least 49 days after the endpoint of treatment (**Figure 5B**). This study reports, for the first time, experimental evidence of the prolonged analgesic effect of cumulative EA treatment in an orofacial persistent pain model induced by MDPI.

To further support our results, we report molecular evidence of various mechanisms associated with persistent orofacial pain that were effectively modulated for at least 49 days after the end of cumulative EA treatment. For instance, mounting evidence suggests that the analgesic effects of EA may be facilitated by the inhibition of glial cells (37). Similarly, our previous study demonstrated that cumulative EA treatment effectively relieved dental pain and inhibited microglial and astrocyte activation (16). In addition, recent evidence has reported that cumulative EA treatment reversed GLAST and GLT-1 downregulation following spared nerve injury (SNI), suggesting the involvement of glutamate transporter modulation in EA analgesic effects (101). In accordance with this, we observed prolonged astrocyte inhibition and longlasting GLT-1 and GS upregulation in the Vi/Vc transition zone of MDPI+EA mice (**Figures 6A–C**). Moreover, EA has been demonstrated to prevent neuronal apoptosis by downregulating proapoptotic proteins (cleaved caspase-3, 9 and cleaved-PARP) and facilitating the upregulation of the anti-apoptotic protein Bcl-2 following SCI (102). Furthermore, novel experimental evidence suggests that EA may regulate the GABAergic system via cannabinoid receptor type 1 (CB1) of the ventrolateral periaqueductal gray matter (vlPAG) and induce analgesia (103). In agreement with these previous reports, we observed an extended regulation of apoptotic markers such as cleaved caspase-3, Bax, and Bcl-2 (**Figures 7A, B**) and the prolonged upregulation of the GAD65 isoform of GABAergic interneurons in the Vi/Vc transition zone of MDPI+EA mice (**Figures 8A–C**). However, we strongly suggest that the mechanism mentioned above is associated with the prolonged cumulative analgesic effects of EA. We hypothesized that a specific central or peripheral mechanism would play an important role in the long-term analgesic effect of cumulative EA treatment.

This study has reported for the first time experimental evidence that cumulative EA treatment exerts prolonged analgesic effects by combining the long-term modulation of multiple signaling pathways involving astrocytes, apoptotic pathways, and GABAergic inhibitory interneuron plasticity in a persistent orofacial pain model induced by MDPI.

## Conclusions

Our results suggest that persistent dental pain in mice can be induced by MDPI. This effect is associated with various trigeminal nociceptive mechanisms, including GABAergic interneuron plasticity and morphological and functional changes in astrocytes. Moreover, EA provides prolonged analgesic and neuroprotective effects that might be associated with the modulation of neuron-glia crosstalk mechanisms. Our findings shed light on persistent dental pain neuromodulation and provide insights into possible alternative pain management treatments using EA therapy.

## Data availability statement

The original contributions presented in the study are included in the article/**Supplementary Material**. Further inquiries can be directed to the corresponding authors.

## Ethics statement

The animal study was approved by China Medical University Institutional Animal Care and Use Committee following the Care and Use of Laboratory Animal Guidebook issued by the Chinese Taipei Society of Laboratory Animal Sciences (CMUIACUC-2021-270). The study was conducted in accordance with the local legislation and institutional requirements.

## Author contributions

All experiments were designed by SR, Y-HC, SY-H, S-YC, and L-JF and performed by SR; SR, S-YC, and Y-CH analyzed the data; SR and S-YC generated the figures; SR and Y-HC wrote the manuscript; Y-HC, Y-CL and S-YH critically revised the manuscript. All the authors have read and agreed to the published version of the manuscript.
