# Supplementary material for: Electroacupuncture exerts prolonged analgesic and neuroprotective effects in a persistent dental pain model induced by multiple dental pulp injuries: GABAergic interneurons-astrocytes interaction
Source: Front Immunol. 2023 Oct 26;14:1213710. doi: 10.3389/fimmu.2023.1213710 (PMC10639134; doi:10.3389/fimmu.2023.1213710)
Supplement: Supplementary file 1 [file DataSheet_1.docx]

**Supplementary Material for**

# Electroacupuncture exerts prolonged analgesic and neuroprotective effects in a persistent dental pain model induced by multiple dental pulp injuries: GABAergic interneurons-astrocytes interaction

Sharmely Sharon Ballon Romero, Lih-Jyh Fuh, Shih-Ya Hung, Yu-Chen Lee, Yu-Chuen Huang, Szu-Yu Chien and Yi-Hung Chen

**1. Supplementary Figure 1**

A pilot study in female mice was conducted to evaluate a possible HWT sex differences following SDPI and MDPI (Fig. S1A). At baseline measurements, all three experimental groups, i.e. control, SDPI and MDPI, showed similar HTW of approximately 25 g (Fig. S1A). From days one to 70, both female mice groups, SDPI or MDPI, showed a significant and persistent decrease in HWT when compared to the control values (Fig. S1A). Furthermore, we also evaluated the body weight loss female mice study following SDPI or MDPI. Baseline evaluations showed a comparable body weight between all experimental groups of approximately 25g (Fig. S1B). On days minus six and four, MDPI female mice that underwent the first pulp exposure exhibited a significant reduction in body weight compared to the controls (Fig. S1B). There was no significant difference in body weight between the SDPI and control groups (Fig. S1B). On days one and three, female mice that experienced a second pulp exposure (MDPI) or SDPI, and control showed a considerable body weight loss, and there was no significant difference between them (Fig. S1B). From day seven until the end of our evaluations at 70 days, all three female experimental groups started to regain weight (Fig. S1B).





**Fig. S1. Nociceptive thresholds and body weight measurement in female mice following SDPI or MDPI.**

**(A)** Mechanical sensitivity in female mice measured by the von Frey test. On day one dramatic changes in head withdrawal thresholds (HWT) in SDPI or MDPI female mice. From three to 70 days, persistent reduction in HWT in SDPI or MDPI female animals was found compared to controls. There was no significant difference in HWT at either time point between SDPI or MDPI female mice.

Short-term body weight loss in MDPI but not SDPI female mice. **(B)** Short-term effects of MDPI but not SDPI in female mice body weight. Data are presented as the mean±SEM. Between-group comparisons were performed by repeated-measures ANOVA at each time point, followed by Bonferroni’s *posthoc* test (^*^*p*<0.05, ^**^*p*<0.01, ^***^*p*<0.001 vs. control; ^###^*p*<0.001, vs. SDPI; *n*=9 in each group).
